# Oral and Extra-Oral Manifestations of Hypersensitivity Reactions in Orthodontics: A Comprehensive Review

**DOI:** 10.3390/jfb15070175

**Published:** 2024-06-27

**Authors:** Federica Di Spirito, Alessandra Amato, Maria Pia Di Palo, Rahila Ferraro, Davide Cannatà, Marzio Galdi, Elettra Sacco, Massimo Amato

**Affiliations:** 1 Department of Medicine, Surgery and Dentistry, University of Salerno, Via S. Allende, 84081 Baronissi, SA, Italy; r.ferraro21@studenti.unisa.it (R.F.); davide2897@icloud.com (D.C.); marzio.galdi@gmail.com (M.G.); elettra.sacco@hotmail.it (E.S.); mamato@unisa.it (M.A.); 2 Department of Neuroscience, Reproductive Science and Dentistry, University of Naples Federico II, 80131 Naples, NA, Italy; aaleamato@gmail.com

**Keywords:** hypersensitivity, hypersensitivity reactions, oral lesions, orthodontic appliance, orthodontic treatment, orthodontics, titanium alloy, titanium, nickel, resins, metals

## Abstract

Although rare, oral manifestations of hypersensitivity reactions in orthodontic patients pose a significant clinical challenge due to their heterogeneous presentations, and can cause discomfort and pain, possibly impacting patients’ quality of life and orthodontic treatment duration and outcomes. This comprehensive review aimed to elucidate the oral, perioral, and systemic manifestations of hypersensitivity reactions in orthodontic subjects, focusing on patients with fixed appliances, removable appliances, and clear aligners, and detailing their epidemiology, macroscopic and microscopic features, allergy testing, clinical implications, and specific management strategies. Oral and extra-oral manifestations of (immediate and delayed) hypersensitivity reactions occur rarely and are due to the release of metal and non-metal ions from orthodontic appliances. They typically present as erythema, erosive–ulcerative lesions, and gingival hyperplasia, with histopathological findings showing inflammatory infiltrates. Nickel is a significant allergen, and diagnostic tests like patch tests are essential for managing these reactions. Likely due to prolonged contact with oral tissues, fixed orthodontic appliances pose a higher risk compared to removable appliances and clear aligners. Early identification and removal of allergenic materials, combined with effective treatments, can resolve symptoms and prevent recurrence. Keeping dental and medical records updated and knowing family and personal medical histories helps clinicians choose appropriate materials and counsel patients about potential risks. Proper patient education, regular monitoring, and using hypoallergenic materials are key strategies for managing these reactions.

## 1. Introduction

Oral and extra-oral (perioral and systemic) manifestations of hypersensitivity reactions are adverse inflammatory responses of the oral tissues to various allergens in dental materials, typically metals and polymers [1]. Oral manifestations of hypersensitivity reactions to dental materials are relatively uncommon but can pose significant challenges when they occur. Common manifestations include oral lichenoid lesions, which can appear adjacent to amalgam restorations as white, reticular, or erosive patches on the mucosa, erythema, swelling, unspecified eritematous or erosive–ulcerative lesions, frequently accompanied by burning sensations [2,3]. Similar macroscopic features were recorded for lesions occurring on the lips, perioral skin, hands, and feet, and systemic manifestations were also described [2,3]. Oral lesions related to dental material hypersensitivity typically exhibit microscopic features resembling other contact hypersensitivity reactions. This includes a band-like lymphocytic infiltrate in the superficial lamina propria, basal cell degeneration, and varying degrees of hyperkeratosis. Indeed, the presence of these histological features can help distinguish hypersensitivity reactions from other pathologies, such as autoimmune conditions or infectious processes [4].

Hypersensitivity reactions are triggered when the immune system overreacts to specific allergens present in dental materials. While their epidemiology is relatively low, affecting approximately 0.3–0.4% of patients undergoing dental treatment, with an occurrence rate between 1 in 700 and 1 in 2600 procedures in dental practices [2], they are clinically significant, especially in individuals with a history of atopy or previous sensitization to metals [5]. Indeed, dental patients with these manifestations often have a family or personal history of atopic conditions, such as asthma, allergic rhinitis, and atopic dermatitis; are under treatment with immunosuppressive or anti-inflammatory drugs [2,6]; and report allergies to metals, such as nickel and cobalt, chromium, palladium, and acrylates used in dentures and composites, and other common allergens like latex and certain medications.

Hypersensitivity reactions to dental materials are most frequently reported for metals used in dental amalgams, particularly nickel, palladium, chromium, and cobalt [2,4]. Nickel, in particular, is a major allergen [5]. Notably, nickel hypersensitivity is particularly prevalent in the general population, affecting approximately 10–30% of females and 1–3% of males [7]. Diagnosing hypersensitivity to dental materials involves a combination of clinical examination, patient history, and allergy tests (e.g., patch test, prick tests), confirmed when the removal or replacement of the suspected material leads to resolution or improvement of symptoms. Indeed, the primary treatment for hypersensitivity reactions to dental materials involves the removal and replacement of the offending material. Accordingly, replacing amalgam restorations with alternative materials, such as composites or glass ionomer cements, results in significant clinical improvement [8]. Topical corticosteroids may be used to manage inflammation and symptomatic relief, while in cases of systemic involvement, systemic corticosteroids or immunosuppressive therapies may be necessary [8].

In dental patients, especially those with orthodontic appliances containing nickel, the prevalence of allergic reactions can be significant, likely due to prolonged exposure to nickel-containing appliances [9]. Studies indicate that up to 30% of female and 3% of male orthodontic patients may develop hypersensitivity reactions to nickel [7,10]. This increased risk is often associated with previous sensitization from non-dental sources such as earrings or body piercings [10].

The clinical presentation of the oral manifestations of hypersensitivity reactions can vary, but it usually includes erythema, erosive–ulcerative lesions, and gingival hyperplasia [11], often accompanied by pain, itching, and burning sensations [7]. Therefore, understanding the clinical and histologic features of these reactions, along with effective diagnostic and treatment strategies, is crucial for orthodontic practitioners to ensure appropriate management and prevention of further reactions [10].

Considering that oral manifestations of hypersensitivity reactions in orthodontic patients pose a significant clinical challenge due to their heterogeneous presentations, and can cause discomfort and pain, possibly impacting on patients’ quality of life [12] and orthodontic treatment duration and outcomes, the present comprehensive review aimed to elucidate the oral, perioral, and systemic manifestations of hypersensitivity reactions in orthodontic subjects, with a focus on patients with fixed appliances, removable appliances, and clear aligners, detailing their epidemiology, macroscopic and microscopic features, allergy testing, clinical implications, and specific management strategies.

## 2. Materials and Methods

The authors searched the electronic databases MEDLINE/PubMed, Scopus, and Web of Science for case reports, case series, cross-sectional, case-control, retrospective, and prospective studies as well as randomized clinical trials on epidemiology, on macro/microscopic features and management of oral and extraoral (perioral and systemic) manifestations of hypersensitivity reactions in orthodontic patients treated with removable/fixed appliances or clear aligners, published in English by 28 January 2024.

The following keywords, combined with Boolean operators, were used: adverse reaction OR hypersensitivity OR allergy OR sensitivity OR hypersensitivity reaction OR allergic OR allergic reaction OR sensitive OR sensitivities AND orthodontic OR orthodontic material OR orthodontic appliances OR clear aligners OR aligner OR Invisalign.

A further search delved into the pathogenic mechanisms underlying hypersensitivity reactions, the therapeutic approach, and the general management of the manifestations studied.

The references were managed with the Mendeley Reference Manager tool version 2.80.1.

## 3. Results

**Figure 1:**
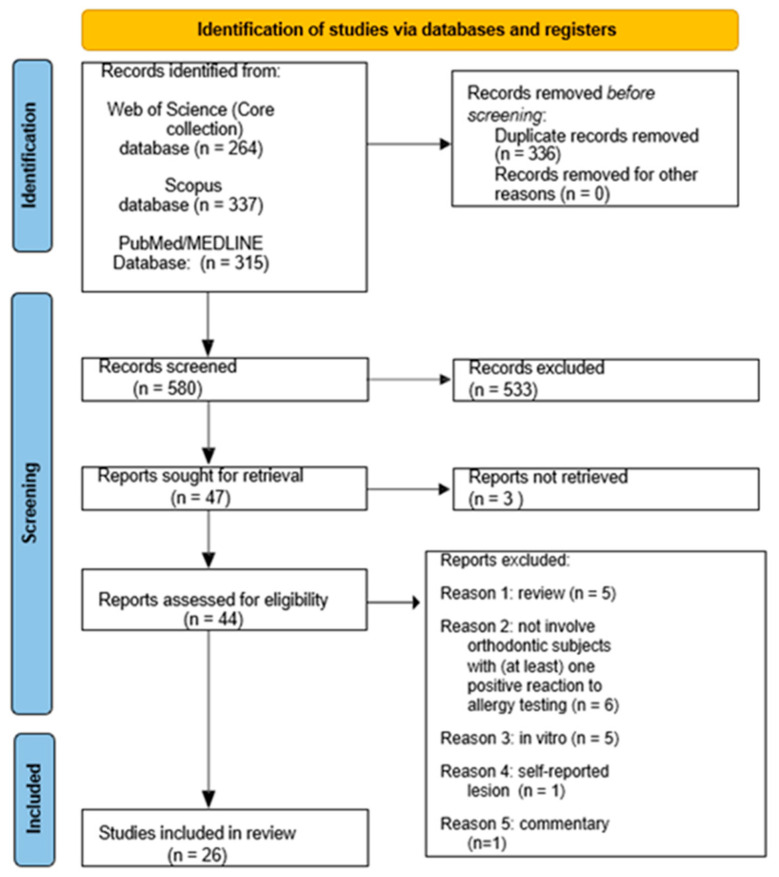
Flow-chart for study selection of case reports, case series, cross-sectional, case-control, retrospective and prospective studies as well as randomized clinical trials on epidemiology, on macro/microscopic features and management of oral and extraoral (perioral and systemic) manifestations of hypersensitivity reactions in orthodontic patients treated with removable/fixed appliances or clear aligners, published in English by 28 January 2024.

The study selection of records identified via databases was presented in Figure 1.

## 4. Orthodontic Hypersensitivity Reactions

### 4.1. Risk Factors

**Figure 2:**
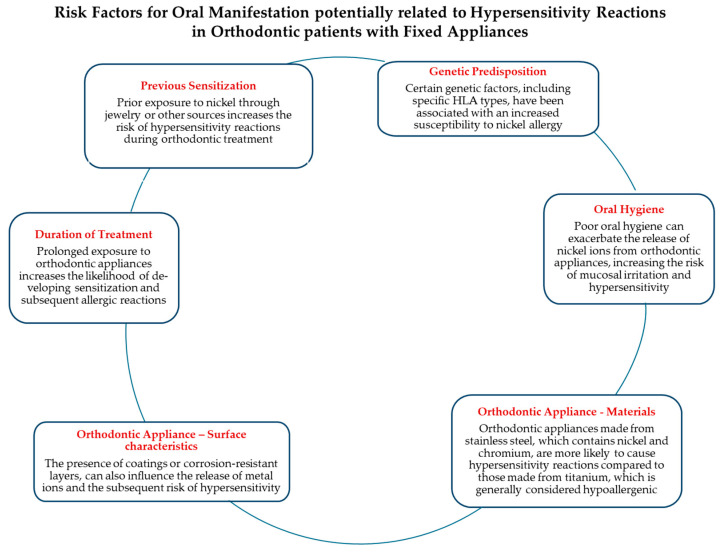
Risk factors for oral manifestation of hypersensitivity reactions in orthodontic patients with fixed appliances.

**Figure 3:**
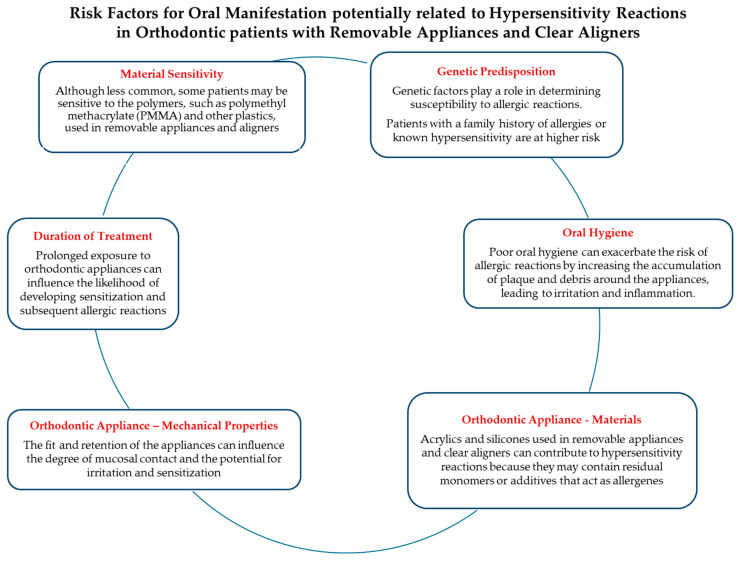
Risk factors for oral manifestation of hypersensitivity reactions in orthodontic patients with removable appliances and clear aligners.

Several patient-related systemic and local factors [13,14], as well as factors related to the orthodontic appliance [15], have been implicated in the development of hypersensitivity reactions and associated oral manifestations in orthodontic patients with fixed appliances (Figure 2), removable appliances, and clear aligners (Figure 3).

### 4.2. Ions Release from Orthodontic Appliances

The release of metal and non-metal ions from orthodontic appliances significantly contributes to hypersensitivity reactions, both immediate and delayed.

In vitro studies, typically employing artificial saliva to replicate the oral environment and measuring the concentration of metal ions released over time, have been instrumental in understanding the corrosion behavior and ion release from orthodontic materials under simulated oral conditions.

Research has shown that nickel and chromium ions are released from stainless steel brackets and wires when exposed to conditions mimicking the oral cavity, and that the amount of ion release is influenced by factors such as the pH of saliva, the presence of fluoride, and the type of orthodontic material used. For instance, studies have demonstrated that acidic environments can significantly increase nickel release from stainless steel brackets, highlighting the role of oral pH in metal ion release [16].

Further in vitro investigations into nickel–titanium (NiTi) alloys, commonly used in orthodontic wires, revealed that nickel release is higher during the initial stages of immersion in artificial saliva but decreases over time as a passive oxide layer forms on the alloy surface. This oxide layer serves as a protective barrier, reducing further ion release and enhancing the biocompatibility of the material [15].

In vivo studies, monitoring ion levels in patients’ saliva, blood, or urine over time during orthodontic treatment, provide a more comprehensive understanding by considering the dynamic interactions within the oral cavity, including saliva, food, and microbial activity. Research has indicated that concentrations of nickel, chromium, and zinc ions are significantly higher in the saliva of patients with fixed orthodontic appliances compared to those without such appliances, indicating active ion release in the oral environment [17].

Moreover, systemic absorption of nickel and chromium from fixed orthodontic appliances has been demonstrated by measuring these metals in blood and urine samples. Elevated levels of these ions suggest that they can be absorbed systemically, potentially contributing to hypersensitivity reactions [15].

Notably, in vivo studies also underscore the impact of oral hygiene on ion release, with poor oral hygiene leading to increased corrosion of orthodontic materials and, consequently, higher risks of hypersensitivity reactions [14].

### 4.3. Immediate Hypersensitivity Reactions

Immediate hypersensitivity reactions, also known as Type I hypersensitivity reactions, are mediated by IgE antibodies and occur rapidly upon exposure to an allergen. In orthodontic patients, these reactions can be triggered by various materials used in dental appliances, such as metals, latex, and certain polymers used in removable appliances and clear aligners. Understanding these reactions is critical for preventing and managing adverse effects in susceptible individuals [13].

During the initial sensitization phase, the allergen (e.g., nickel ions, latex proteins) enters the body and is recognized by antigen-presenting cells, such as Langerhans cells. These cells process the allergen and present it to T-helper cells, which subsequently stimulate B-cells to produce IgE antibodies specific to the allergen. The IgE antibodies then bind to the surface of mast cells and basophils, sensitizing these cells to the allergen [13].

Upon subsequent exposure to the same allergen, the allergen cross-links the IgE antibodies on the surface of the sensitized mast cells and basophils, leading to their degranulation and the release of various mediators, including histamine, prostaglandins, and leukotrienes. These mediators cause the clinical manifestations of immediate hypersensitivity reactions, such as urticaria, angioedema, bronchospasm, and anaphylaxis [13].

### 4.4. Delayed Hypersensitivity Reactions

The mechanism underlying nickel hypersensitivity involves a type IV delayed hypersensitivity reaction, which is mediated by T-cells.

The release of metal ions, such as nickel, cobalt, and chromium, from orthodontic appliances can trigger a cascade of immune responses [18]. These metal ions penetrate the skin or mucosa [16] and act as haptens, binding to proteins and forming complexes that are recognized as foreign by the immune system. This process initiates a sensitization phase, where antigen-presenting cells such as Langerhans cells capture and process the metal–protein complexes. These cells then migrate to regional lymph nodes, presenting the processed antigens to naive T-cells, which then proliferate and differentiate into memory T-cells [15].

Upon subsequent exposure to the same metal ions, these memory T-cells recognize the antigen and mount a rapid immune response. This effector phase involves the release of cytokines and chemokines that recruit other immune cells, such as macrophages and eosinophils, to the site of exposure. This results in a localized inflammatory response characterized by erythema, edema, and sometimes ulceration [15].

In addition to T-cell-mediated responses, there is evidence that B-cells and antibodies may also play a role in some cases of metal hypersensitivity [13].

The presence of specific IgE antibodies against metal ions has been detected in some patients, suggesting a potential for immediate hypersensitivity reactions in addition to delayed-type reactions [13].

## 5. Orthodontic Materials Associated with Hypersensitivity Reactions

The evidence of hypersensitivity to various orthodontic materials, the most important of which are described below, emphasizes the need for careful assessment and management of patients undergoing orthodontic treatment.

### 5.1. Nickel

Nickel is one of the most common allergens in orthodontics, primarily due to its widespread use in stainless steel brackets and wires. Studies indicate that the prevalence of nickel hypersensitivity ranges from 10% to 30% in the general population, with higher rates observed in females. In orthodontic patients, the prevalence of nickel hypersensitivity varies but is significant, particularly among those with a prior history of nickel allergy from jewelry or other exposures [14].

The epidemiological studies on nickel hypersensitivity have consistently shown a higher prevalence in females, often linked to earlier and more frequent exposure to nickel through body piercings and jewelry [10]. According to a study by Kerosuo et al. (1996), the prevalence of nickel allergy in adolescents was found to be 30% in girls and 3% in boys, highlighting the gender disparity in hypersensitivity reactions [17]. This disparity is also reflected in the orthodontic context, where female patients exhibit higher rates of allergic reactions to nickel-containing appliances [10].

There is evidence to suggest geographical and ethnic variations in the prevalence of nickel hypersensitivity. Studies conducted in different regions have reported varying prevalence rates, which could be attributed to genetic factors, cultural practices (such as the prevalence of ear piercing), and environmental exposures [5]. For instance, a study conducted in Denmark reported a prevalence of 10% among the general population, with higher rates observed in individuals with multiple piercings [15]. A study conducted in Sweden found a significant correlation between lifestyle factors, such as ear piercing, and the prevalence of nickel allergy in adolescents [19].

Remarkably, nickel hypersensitivity is not only a localized issue but can also trigger systemic reactions [20,21]. For instance, patients with nickel hypersensitivity may experience exacerbation of conditions like eczema upon exposure to nickel-containing orthodontic appliances [5,22]. This broadens the clinical implications of nickel hypersensitivity, necessitating comprehensive patient education and preventive strategies.

### 5.2. Other Metals

In addition to nickel, other metals used in orthodontics, such as cobalt, chromium, manganese, and titanium, can also cause hypersensitivity reactions [23]. The prevalence of hypersensitivity to these metals is generally lower than that to nickel but can still pose significant clinical challenges [24].

For instance, cobalt and chromium are often components of stainless steel and can elicit allergic responses in sensitized individuals [13].

Titanium, commonly used in implants, is generally considered to be biocompatible, but there have been reports of hypersensitivity reactions in some cases [2].

Awareness of possible hypersensitivity reactions to these metals is particularly important in patients with a history of allergies or dermatitis. Indeed, in patients with a known allergy to chromium, avoiding stainless steel devices containing this metal is recommended [13]. In addition, the development of metal-free alternatives such as ceramic brackets and titanium nitride-coated wires has been recommended to reduce these risks [2,25].

### 5.3. Acrylics and Polymers

Acrylics and polymers are commonly used in removable appliances and clear aligners. Hypersensitivity reactions to these materials are less frequent compared to metals like nickel but can still occur [5]. The prevalence of hypersensitivity to acrylics is estimated to be lower, ranging from 0.1% to 1% in the general population, depending on the specific composition of the materials and the individual patient’s sensitivity [26].

Although the incidence of hypersensitivity to these materials is lower, clinicians should remain aware of the potential for allergic reactions and be prepared to manage them appropriately [5]. Enhanced manufacturing techniques and material formulations are being explored to reduce the potential for these monomers to leach out [19].

### 5.4. Composite Resins

Composite resins used in bonding, beyond restorative procedures, can also cause hypersensitivity reactions, particularly due to components like bisphenol A-glycidyl methacrylate (Bis-GMA). The prevalence of hypersensitivity to composite resins is relatively low but noteworthy, especially in patients with a history of multiple chemical sensitivities [10].

Photoinitiators, such as camphorquinone, and other additives in composite resins can also contribute to hypersensitivity reactions [2]. Therefore, understanding the complete chemical composition of these materials is essential for predicting and managing potential hypersensitivity reactions.

### 5.5. Latex

Latex hypersensitivity is another common concern in orthodontics, particularly given its use in gloves, elastics, and other dental products.

The prevalence of latex allergy is estimated to be around 1% to 6% in the general population, with higher rates in healthcare workers and individuals with multiple surgeries [27]. In orthodontic patients, latex hypersensitivity can manifest as contact dermatitis or, in severe cases, as systemic reactions such as anaphylaxis [27]. In light of this, the use of latex-free alternatives in dental and orthodontic practices to prevent allergic reactions, such as nitrile gloves and non-latex elastics, which have been shown to be effective and safe substitutes [26], should be encouraged [19].

## 6. Oral Manifestations of Hypersensitivity Reactions in Orthodontic Patients

### 6.1. Epidemiology

This chapter delves into the epidemiological aspects of oral manifestations of hypersensitivity reactions in orthodontic patients, highlighting the prevalence associated with different types of orthodontic appliances.

#### 6.1.1. Fixed Orthodontic Appliances

The prevalence of oral manifestations of hypersensitivity reactions among orthodontic patients is estimated to be around 0.1–0.2%, indicating a relatively low but noteworthy incidence [28,29,30].

However, patients with fixed appliances are considered at a significantly higher risk of developing hypersensitivity reactions compared to removable ones due to the prolonged and consistent contact with various materials such as metals and adhesives used in these devices [29,30].

The higher incidence observed in females is attributed to prior sensitization from nickel-containing jewelry, which primes the immune system to respond more vigorously upon subsequent exposures [7]. Indeed, nickel, also present in stainless steel and nickel–titanium alloys used in orthodontics, is a well-known allergen that can trigger both localized and systemic hypersensitivity reactions [31].

The mean age of affected patients is typically in the adolescent range, coinciding with the age group most commonly undergoing orthodontic treatment [14].

The duration since the beginning of treatment is a critical factor, as prolonged exposure increases the likelihood of developing sensitization and subsequent hypersensitivity reactions [14].

#### 6.1.2. Removable Orthodontic Appliances and Clear Aligners

The incidence of oral manifestations of hypersensitivity reactions in orthodontic patients with removable appliances is lower compared to those with fixed appliances [32], although the gender ratio remains consistent with the patterns observed with fixed appliances [33], with a higher incidence in females [34]. However, studies have shown that even non-metallic materials can cause allergic reactions in susceptible individuals, particularly with prolonged use and inadequate oral hygiene [35].

Clear aligners made from various polymers can also induce oral manifestation of hypersensitivity reactions [36], though less frequently than metal-based fixed appliances, mainly due to the absence of nickel and other metals commonly implicated in allergic reactions [26].

### 6.2. Clinical/Macroscopic Features

Erythema, characterized by redness and inflammation, of the mucosal tissues is a common initial sign of hypersensitivity, and is often localized to the areas in direct contact with the orthodontic materials, such as brackets and wires [37].

Erosive–ulcerative lesions usually develop as the inflammatory response progresses, and are generally surrounded by an erythematous halo. They are typically painful and can significantly impair oral functions, such as eating and speaking [38].

Gingival hyperplasia, or gingival overgrowth, is another prominent feature of hypersensitivity reactions in orthodontic patients. This condition can result from the chronic irritation and inflammatory response induced by orthodontic appliances. The hyperplastic tissue often appears swollen and may bleed easily, complicating oral hygiene and potentially leading to secondary infections [7].

Mucosal desquamation is generally a severe manifestation of hypersensitivity. This condition exposes the underlying tissues, leading to increased sensitivity and risk of infection. Patients experiencing mucosal desquamation often report significant discomfort and may require immediate intervention to prevent further complications [10].

Erythema, ulceration, and lichenification often occur together, accompanied by symptoms such as burning, pain, itching, and paresthesia [39], and overlap with other inflammatory mucosal conditions, which complicates their evaluation and treatment and together configure the picture of allergic contact stomatitis [39].

### 6.3. Histopathologic/Microscopic Features

The histopathologic examination of oral lesions resulting from hypersensitivity reactions, although rarely performed, provides critical insights into the underlying pathophysiology. Microscopy often reveals inflammatory infiltrates, predominantly composed of lymphocytes, and eosinophils, indicative of an immune-mediated hypersensitivity response [7].

The presence of lymphocytes, specifically T-helper 1 cells [39], in the infiltrates suggests a type IV hypersensitivity reaction, which is cell-mediated and involves T-cell activation [4].

Eosinophils, on the other hand, are typically associated with type I hypersensitivity reactions, which are IgE-mediated. The co-existence of these cell types in the inflammatory infiltrate highlights the complex nature of hypersensitivity reactions in the oral cavity, where both immediate and delayed immune responses can be at play [40].

Microscopic findings often include significant edema, or swelling, within the mucosal tissues, resulting from increased vascular permeability [41].

Additionally, vascular changes such as dilation and congestion are common, reflecting the inflammatory process’s impact on blood vessels. These changes contribute to the clinical appearance of erythema and swelling observed in hypersensitivity reactions [7].

### 6.4. Diagnosis

The diagnosis of oral manifestations of hypersensitivity reactions is based on a combination of clinical presentation and histological findings.

A thorough clinical evaluation is the first step, focusing on the patient’s history of exposure to potential allergens [42], such as metals in orthodontic appliances, systemic drugs, or recent vaccinations [10,43]. Detailed documentation of symptoms, their onset, and progression helps in correlating them with possible triggers [10].

Histopathological examination of biopsy specimens can confirm the diagnosis by revealing characteristic features of hypersensitivity reactions, such as inflammatory infiltrates and tissue changes. This step is particularly useful in distinguishing hypersensitivity reactions from other oral pathologies that may present with similar clinical features.

Allergy tests, which are described later, aim to identify the specific allergen responsible for the hypersensitivity reaction. A positive reaction helps in pinpointing the allergen, correlates the manifestation with the hypersensitivity reaction to the specific allergen, and enables targeted treatment strategies [40].

### 6.5. Treatment

Effective management of oral manifestations of (ascertained) hypersensitivity reactions in orthodontic patients involves removing the offending agent and addressing the symptoms through pharmacological and non-pharmacological means.

Identifying and removing the allergenic material is the cornerstone of managing hypersensitivity reactions [44]. In orthodontic patients, this may involve replacing nickel-containing appliances with hypoallergenic alternatives such as stainless steel or titanium brackets [10].

The pharmacological treatment usually includes the administration of topical corticosteroids, commonly used to reduce inflammation and alleviate symptoms, applied directly to the affected areas to minimize systemic side effects and provide targeted relief [7] and systemic corticosteroids, prescribed in severe cases, for short periods to prevent long-term side effects to control the widespread inflammatory response. These medications used [40], as well as antihistamines, are beneficial in managing symptoms associated with type I hypersensitivity reactions by blocking histamine release and reducing allergic symptoms such as itching and swelling.

Non-pharmacological treatment generally comprises good oral hygiene maintenance, helping in reducing secondary infections and promoting healing of the affected mucosal tissues [29,30,45], and dietary modifications to avoid foods that exacerbate oral symptoms, while opting for non-irritating foods is recommended to minimize further mucosal irritation. Patients should also be educated about the importance of avoiding known allergens [39].

### 6.6. Progression

The progression of oral manifestations of hypersensitivity reactions varies depending on the severity of the reaction and the timeliness of intervention, and the long-term outlook for patients with oral manifestations of hypersensitivity reactions is generally favorable with appropriate management.

In many cases, lesions resolve rapidly upon removal of the offending agent. The inflammatory response subsides, leading to a reduction in symptoms such as erythema, swelling, and discomfort. Regular follow-up is essential to ensure complete resolution and to monitor for any signs of recurrence [7].

However, some cases may require prolonged management, particularly if the hypersensitivity reaction is severe or if there has been significant tissue damage. Chronic lesions may necessitate ongoing treatment with topical or systemic medications to control inflammation and promote healing [29,30]. Patients with persistent or recurrent lesions may require ongoing management strategies, including periodic follow-ups and adjustments to their treatment plans to ensure an optimal outcome [39,46].

Moreover, preventing recurrence involves a combination of strategies, including patient education, the use of hypoallergenic materials, and regular monitoring. Patients should be informed about the importance of avoiding known allergens and maintaining good oral hygiene. Regular follow-up visits allow for early detection and management of any new or recurring symptoms [40].

### 6.7. Characteristics of the Orthodontic Patients

Understanding the characteristics of orthodontic patients with oral manifestation of hypersensitivity reactions is crucial for effective management and prevention. This chapter explores the common comorbidities, ongoing pharmacological treatments, and the significance of personal and family histories of allergies among these patients.

#### 6.7.1. Gender

Females are more frequently affected by hypersensitivity reactions to orthodontic materials, such as nickel, primarily due to their higher likelihood of prior sensitization through exposure to nickel-containing jewelry and other sources [7]. The increased prevalence in females is also influenced by hormonal factors that can exacerbate immune responses [29].

This predisposition results in a more vigorous immune response upon subsequent exposure during orthodontic treatment [29].

#### 6.7.2. Age

Adolescents are the group most commonly affected by hypersensitivity reactions during orthodontic treatment. This is largely due to the fact that this age group is the primary demographic undergoing orthodontic procedures. The incidence of hypersensitivity reactions is notably higher in this population because of the extended duration of treatment and continuous exposure to potential allergens such as nickel and chromium in fixed appliances [14,47].

Furthermore, younger patients may have more reactive immune systems, which can lead to more pronounced hypersensitivity reactions compared to older individuals.

#### 6.7.3. Comorbidities

Patients with oral manifestations of hypersensitivity reactions often present with a range of comorbidities that can predispose, exacerbate, and complicate their management, and are typically characterized by an overactive immune response, which can heighten the likelihood and severity of hypersensitivity reactions to orthodontic materials [40].

Atopic dermatitis, a chronic inflammatory skin condition, is frequently associated with other atopic diseases, including asthma and allergic rhinitis. Patients with atopic dermatitis have a defective skin barrier, making them more susceptible to allergens and irritants. This increased permeability can lead to heightened immune responses when exposed to allergens present in orthodontic materials, such as nickel or latex [7].

Asthma, another common comorbidity, is characterized by chronic inflammation of the airways. The inflammatory mediators involved in asthma can also play a role in hypersensitivity reactions, as systemic inflammation can potentiate localized allergic responses in the oral cavity. This relationship underscores the importance of managing these underlying conditions to mitigate the severity of hypersensitivity reactions during orthodontic treatment [40].

Patients with a history of other allergic conditions, such as hay fever or food allergies, are also at increased risk of developing hypersensitivity reactions to orthodontic materials. These patients often have a heightened immune response to various allergens, which can include the metals and polymers used in orthodontic appliances. This predisposition necessitates a thorough pre-treatment assessment to identify and mitigate potential risks [10].

In a recent study of 687 patients reporting adverse effects from dental materials in general, comorbid conditions such as diabetes mellitus, mental and behavioral disorders, and diseases of the musculoskeletal system were also identified as significant contributors to hypersensitivity reactions [48]. These comorbidities often overlap, complicating the clinical picture and management strategies [48].

#### 6.7.4. Ongoing Pharmacological Treatment

The overall management of hypersensitivity reactions in orthodontic patients must consider the potential impact of ongoing pharmacological treatments. Indeed, certain medications, particularly nonsteroidal anti-inflammatory drugs (NSAIDs) and antibiotics, can exacerbate hypersensitivity reactions. Therefore, understanding the patient’s medication history is crucial for tailoring treatment plans that minimize the risk of adverse reactions [49].

NSAIDs are commonly used to manage pain and inflammation but can also trigger or worsen hypersensitivity reactions. These medications can inhibit cyclooxygenase enzymes, leading to an imbalance in the production of prostaglandins and leukotrienes, which are key mediators in inflammatory and allergic responses. Patients taking NSAIDs may exhibit more severe symptoms when exposed to orthodontic materials that they are allergic to [7].

Certain antibiotics, particularly those in the penicillin and cephalosporin classes, are known to cause hypersensitivity reactions. These reactions can range from mild rashes to severe anaphylaxis. In orthodontic patients, the concurrent use of these antibiotics can amplify the immune response to allergens present in dental materials, leading to more pronounced oral manifestations [40,50]

Psychotropic medications, including antidepressants and antipsychotics, can also contribute to oral hypersensitivity reactions. These drugs may alter the immune response or interact with other medications, increasing the risk of adverse reactions to orthodontic materials [51].

Other medications that can influence hypersensitivity reactions include antihypertensives, antiepileptics, and certain biologics used for autoimmune conditions. These drugs can modulate the immune system in ways that might increase susceptibility to allergic reactions [7].

#### 6.7.5. Family and Personal History of Allergy

A comprehensive history of allergies is critical for identifying patients at risk of hypersensitivity reactions. This history should include personal and familial allergic conditions, previous allergic reactions to metals, medications, and other allergens [10,40].

A familial history of allergies can indicate a genetic predisposition to hypersensitivity reactions. Accordingly, conditions such as atopic dermatitis, asthma, and hay fever often run in families, and this predisposition can extend to reactions against orthodontic materials. Coherently, patients with a family history of atopic diseases were more likely to report adverse effects from dental materials [48]. For this reason, understanding the family history can guide clinicians in choosing hypoallergenic materials and in counseling patients about their potential risks [40]

Patients with a personal history of allergies are more likely to develop hypersensitivity reactions to orthodontic materials. Since allergies to metals, particularly nickel, are common and can result in significant oral manifestations when patients are exposed to nickel-containing orthodontic appliances, identifying specific allergens that the patient has reacted to in the past is crucial [52].

As later described, common allergens in orthodontics include nickel, latex, and certain polymers used in clear aligners and removable appliances [53]. Accordingly, patients should be specifically asked about their reactions to jewelry, latex gloves, and any previous dental materials used in their treatments. This detailed history can inform the selection of alternative materials that are less likely to trigger hypersensitivity reactions [10]. In addition, detailed questioning about past allergic reactions, including the nature and severity of the reactions, helps in predicting and preventing potential hypersensitivity issues [10].

## 7. Allergy Testing

Various diagnostic tests, including patch tests, prick tests, blood tests, and histopathological examinations, play a crucial role in identifying hypersensitivity reactions.

### 7.1. Methods

Allergy testing methods, including patch tests, skin allergy tests, blood tests, and basophil activation tests, beyond histopathological examination, offer comprehensive diagnostic tools to identify and manage allergic conditions.

Each method, synthesized in Table 1, has unique applications, advantages, and limitations, and the choice of test depends on patient-specific factors and clinical scenarios.

#### 7.1.1. Patch Testing

Patch testing is particularly valuable in diagnosing delayed-type hypersensitivity reactions, which are mediated by T-cells and typically manifest 48–72 h after exposure to the allergen. This method helps in identifying specific allergens responsible for allergic contact dermatitis [5].

Patch testing involves applying small amounts of potential allergens to the skin, usually on the back, and covering them with adhesive patches. These patches remain in place for 48 h, after which they are removed, and the skin is examined for reactions at 48 h and again at 72 to 96 h. A positive test is indicated by erythema, edema, or vesiculation at the test site.

Additionally, patch testing can be customized using dental materials, including orthodontic brackets, wires, and elastics, to directly assess the patient’s sensitivity to these specific substances [2]. Accordingly, this test is the gold standard for diagnosing contact hypersensitivity reactions to orthodontic materials [10].

#### 7.1.2. Prick Testing

Prick testing is beneficial for identifying IgE-mediated allergic reactions, such as those caused by latex or certain metals. It is a rapid and minimally invasive test that provides immediate results, making it useful in clinical settings where quick diagnosis is necessary [19].

It involves placing a drop of the allergen on the skin, followed by a gentle prick through the drop into the epidermis. This test is read within 15 to 20 min, and a positive result is indicated by a wheal and flare reaction.

Prick testing is less commonly used for diagnosing hypersensitivity to orthodontic materials but can be helpful in specific cases where immediate reactions are suspected [40].

#### 7.1.3. Blood Tests

Blood tests, including specific IgE antibody tests, are used to detect type I hypersensitivity reactions [58]. These tests measure the levels of IgE antibodies specific to various allergens in the blood. Specifically, the Radioallergosorbent Test (RAST) or ImmunoCAP can quantify specific IgE antibodies, providing valuable information about the patient’s allergic status, and are particularly useful for patients with extensive dermatitis, where skin testing might exacerbate the condition [5].

While less common than skin tests, blood tests can be useful for patients who cannot undergo skin testing due to severe dermatitis or other contraindications [7].

#### 7.1.4. Histopathological Examination

Histopathological examination of biopsy specimens from affected oral tissues can confirm the diagnosis of hypersensitivity reactions. This examination typically reveals inflammatory infiltrates with lymphocytes and eosinophils, indicative of a hypersensitivity response.

This method is particularly useful for confirming the diagnosis of delayed-type hypersensitivity reactions [7], providing definitive evidence of hypersensitivity reactions by identifying characteristic cellular changes. It is especially useful in complex cases where clinical and patch test results are inconclusive [2].

### 7.2. Timing of the Allergy Testing in Relation to the Beginning of Orthodontic Treatment

The timing of allergy testing is critical in preventing and managing hypersensitivity reactions effectively.

#### 7.2.1. Pre-Treatment Testing

Pre-treatment testing is essential for patients with a known history of allergies. Conducting patch tests or specific IgE tests before the initiation of orthodontic treatment can help identify potential allergens and allow clinicians to select hypoallergenic materials.

This proactive approach can prevent the development of hypersensitivity reactions and ensure a smoother treatment process [10].

#### 7.2.2. Testing during Treatment

In many cases, hypersensitivity reactions are not identified until after the onset of symptoms. When patients present with symptoms such as erythema, swelling, or ulceration, patch testing is typically performed to determine the causative allergen.

The timing of these tests performed during the course of treatment can vary but is crucial for managing and mitigating adverse reactions [40].

#### 7.2.3. Post-Treatment Testing

Post-treatment testing may be necessary for patients who develop delayed hypersensitivity reactions after the removal of orthodontic appliances [59].

These tests help confirm the diagnosis and guide future dental treatments, ensuring that materials that cause reactions are avoided in subsequent treatments [7].

## 8. Extra-Oral Manifestations of Hypersensitivity Reactions in Orthodontic Patients

### 8.1. Perioral Manifestations of Hypersensitivity Reactions in Orthodontic Patients

Perioral manifestations of hypersensitivity reactions are fortunately not so common in orthodontic patients. They can in some cases affect the patient’s quality of life and require careful management to avoid complications.

#### 8.1.1. Epidemiology

Perioral manifestations of hypersensitivity reactions are more frequently reported in patients with fixed orthodontic appliances.

The gender ratio is skewed towards females [60], with the mean age of affected individuals being in the adolescent to young adult range [26].

Notably, the mean duration since the beginning of orthodontic treatment correlates with the development of these reactions, with longer treatment durations posing a higher risk [14].

Conversely, subjects undergoing orthodontic treatment with removable appliances and clear aligners experience fewer perioral hypersensitivity reactions. However, when reactions do occur, they follow a similar epidemiological pattern to those with fixed appliances, with a higher prevalence in females and younger patients [33].

#### 8.1.2. Macroscopic and Microscopic Features

The clinical manifestations in patients with removable appliances and clear aligners are similar to those seen with fixed appliances, and include erythema, swelling, and unspecified dermatitis around the lips [61]. In some cases, patients may develop vesicles or blisters, which are often accompanied by itching and discomfort, which can significantly affect the patient’s quality of life, and can rupture and lead to secondary infections if not properly managed [7,26].

Clinical diagnosis involves a thorough patient history and physical examination [62]. Patch testing can be particularly useful in identifying the specific allergens responsible for the reactions. The presence of a positive reaction to nickel or other common allergens confirms the diagnosis and helps in planning appropriate management strategies [10]. For example, perioral dermatitis is often linked to the use of orthodontic headgear containing nickel, which can cause allergic contact dermatitis [63]. This reaction is mediated by T-cells and involves both sensitization and elicitation phases, ultimately leading to skin changes that may necessitate the removal of the offending material [7].

The long-term outcomes of patients with perioral manifestations of hypersensitivity reactions depend on the timely identification and management of the condition. In most cases, the removal of the allergenic material and appropriate medical treatment lead to the resolution of symptoms. However, chronic or recurrent reactions may result in persistent skin changes and discomfort, necessitating ongoing medical care and monitoring [7].

#### 8.1.3. Characteristics of the Orthodontic Patients

Perioral manifestations of potential hypersensitivity reactions are more frequent in patients with a personal or family history of contact dermatitis and allergic rhinitis, increasing the risk of developing perioral reactions [26].

In addition, patients undergoing treatment with specific medications may experience exacerbated perioral reactions. It is crucial to consider these factors when diagnosing and managing hypersensitivity reactions [27].

Furthermore, a history of allergies is a common feature among patients not only with oral but also with perioral manifestations of hypersensitivity reactions. Consequently, detailed patient interviews and clinical vigilance are necessary to identify these risks [10]. Common triggers include nickel and latex found in dental materials, as already discussed [27].

### 8.2. Systemic Manifestations of Hypersensitivity Reactions in Orthodontic Patients

Systemic manifestations of hypersensitivity reactions in orthodontic patients represent a severe and potentially life-threatening clinical scenario; although rare, their severity necessitates prompt recognition and immediate intervention to prevent serious outcomes.

#### 8.2.1. Epidemiology

The prevalence of systemic hypersensitivity reactions to nickel among orthodontic patients is low but significant due to the severe nature of the reactions. Studies have shown that females are more frequently affected than males, which can be attributed to higher rates of sensitization due to nickel exposure from jewelry. Additionally, genetic predisposition plays a crucial role, with certain HLA types being associated with an increased risk of developing nickel hypersensitivity [10].

In subjects undergoing fixed orthodontic treatment, systemic hypersensitivity reactions are rare but more severe, potentially leading to significant clinical complications [64]. The mean age and gender ratio are similar to those observed in perioral reactions, with a higher prevalence in females [65].

In subjects undergoing orthodontic treatment with removable appliances and clear aligners, the incidence of systemic hypersensitivity reactions was lower compared to those with fixed appliances, and reflected similar patterns, with a higher incidence in younger females [33].

#### 8.2.2. Macroscopic and Microscopic Features

Systemic manifestations of hypersensitivity reactions can present with various clinical features, including generalized symptoms such as fever, malaise, and anaphylaxis; the latter obviously requires prompt recognition and treatment [66].

Histologic findings are less specific but can include evidence of systemic inflammation and immune activation [66].

Diagnosis involves a combination of patient history, clinical examination, and diagnostic tests such as skin prick tests, patch tests, and serological assays for specific IgE antibodies.

Management includes immediate discontinuation of the offending material, administration of antihistamines, corticosteroids, and in severe cases, epinephrine.

Long-term management may involve desensitization protocols and the use of alternative medications [40].

#### 8.2.3. Characteristics of the Orthodontic Patients

Patients with systemic hypersensitivity reactions often present with comorbid conditions such as systemic lupus erythematosus or other autoimmune disorders. These conditions can increase the severity of hypersensitivity reactions [66]. Moreover, systemic reactions can be exacerbated by certain medications, necessitating a comprehensive review of the patient’s pharmacological treatment [16].

A history of allergies, including familial predisposition, is a common characteristic of patients with systemic hypersensitivity reactions. This underscores the importance of detailed patient history and clinical assessment [66].

One of the primary culprits for systemic hypersensitivity reactions in orthodontic patients is prolonged exposure to metals such as nickel, which, upon prolonged exposure, can be absorbed into the systemic circulation, leading, in turn, to widespread symptoms such as generalized dermatitis [17], fever, and respiratory distress [16]; other potential include acrylics and composites, and latex [27], as already discussed.

## 9. Discussion

Hypersensitivity to dental materials, especially nickel, remains a significant concern, although its frequency varied across studies, with nickel being a common allergen [5].

Although rare, the epidemiology of hypersensitivity reactions in orthodontics indicates that fixed appliances, often containing nickel and chromium, are more commonly associated with these reactions than removable appliances and clear aligners, likely because the prolonged contact of fixed appliances with oral tissues increases hypersensitivity risk [29]. Nickel, chromium, and certain polymers in orthodontic materials are common triggers for hypersensitivity reactions. However, removable appliances and clear aligners, though less frequently implicated, can still trigger allergic reactions due to materials like polymethyl methacrylate (PMMA) and other polymers [33].

Allergy tests such as patch tests and specific IgE tests are crucial for identifying allergens and guiding treatment. These tests help distinguish between immediate and delayed hypersensitivity reactions, facilitating targeted management strategies [10]. Coherently, treatment usually primarily involves removing the allergenic material, using topical or systemic corticosteroids, and maintaining good oral hygiene [7,10]. However, the progression of hypersensitivity-related oral lesions varies, with many resolving rapidly upon removal of the offending agent, while chronic or severe manifestations may require prolonged management [29,56].

Patients with hypersensitivity reactions often have comorbidities such as atopic dermatitis, asthma, and other allergic conditions. Medications, like NSAIDs and antibiotics, can exacerbate these reactions [40]. Comprehensive histories of personal and familial allergies are crucial for identifying at-risk patients [10]. Therefore, understanding the family history can guide clinicians in choosing hypoallergenic materials and in counseling patients about their potential risks [40]. In addition, detailed questioning about personal history of allergies, including the nature and severity of the past reactions, helps in predicting and preventing potential hypersensitivity issues [10].

Oral manifestations of hypersensitivity reactions in orthodontic patients usually present as localized erythema, erosive–ulcerative lesions, and gingival hyperplasia, severely impacting functions like eating and speaking [7]. The timing of these manifestations varies, with immediate reactions occurring rapidly upon exposure and delayed reactions developing over time [16,40]. Similarly distressing due to their visibility and discomfort, perioral manifestations of hypersensitivity reactions often present as unspecified dermatitis [67], erythema, and swelling around the mouth; though less common, reactions including dermatitis, erythema, and swelling around the lips are often linked to nickel-containing orthodontic headgear [26], and their onset occurs earlier due to direct contact with allergenic materials. Systemic manifestations of hypersensitivity reactions in orthodontic patients, instead, although rare, are severe and include generalized symptoms such as fever, malaise, and anaphylaxis that require immediate medical intervention. These manifestations, which are typically triggered by prolonged exposure to metals such as nickel that can be absorbed systemically [16], can also be influenced by medications and vaccinations administered during orthodontic treatment, especially in patients with a history of allergies [66,68], demonstrating the importance of updating dental and medical records even during orthodontic treatment [7,10,40].

### 9.1. Clinical Insights and Recommendations before and during Orthodontic Treatment for Managing oral Manifestation of Hypersensitivity Reactions in Orthodontic Patients and Comprehensive Orthodontic Patient Care

**Figure 4:**
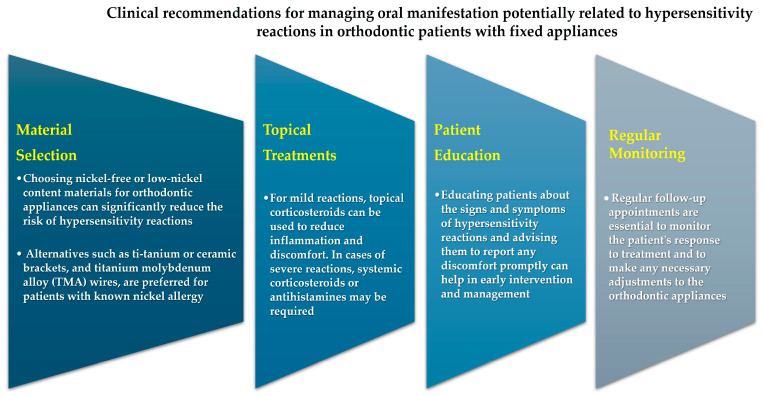
Clinical recommendations for managing oral manifestation of hypersensitivity reactions in orthodontic patients with fixed appliances.

**Figure 5:**
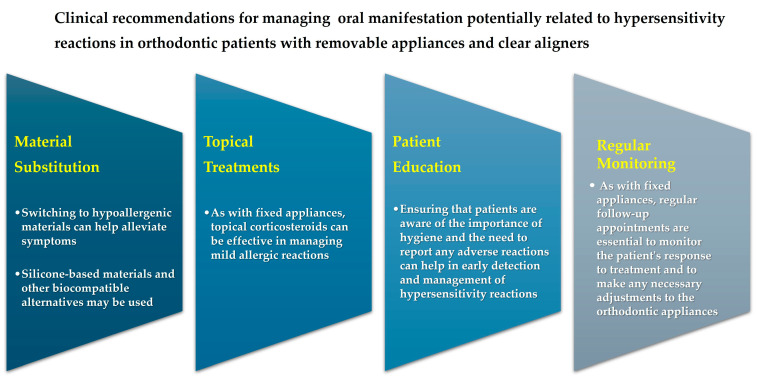
Clinical recommendations for managing oral manifestation of hypersensitivity reactions in orthodontic patients with removable appliances and clear aligners.

Manifestations of hypersensitivity reactions in the oral cavity of orthodontic patients require careful attention and a proactive approach [40]. Through a comprehensive approach, differentiated by device and shown in Figure 4 and Figure 5, that includes patient education, the use of hypoallergenic materials [10], and regular monitoring, clinicians can successfully manage these reactions and ensure optimal outcomes for their patients [40].

Before orthodontic treatment, thorough pre-treatment assessments, including detailed patient history and allergy testing, are crucial for identifying potential allergens.

Allergy testing, such as patch tests or specific IgE tests, helps guide the selection of hypoallergenic materials, such as titanium or ceramic brackets, for patients with a known history of allergies to metals [7].

Patient counseling is also essential to discuss potential risks and alternative treatment options for those with positive allergy test results [10].

Educating patients about the signs and symptoms of hypersensitivity reactions and the importance of maintaining good oral hygiene further aids in prevention [5].

During orthodontic treatment, regular monitoring of patients for any signs of hypersensitivity reactions and early intervention can prevent severe outcomes [69]. Immediate reaction management involves administering antihistamines or corticosteroids, and in cases of severe reactions, epinephrine may be necessary [13]. Removing the offending appliance or material to prevent further exposure is also crucial [14]. Using topical corticosteroids to manage mild reactions and systemic corticosteroids or antihistamines for more severe reactions is vital for effective management [7].

Scheduling frequent follow-up appointments to monitor for any signs of recurring hypersensitivity reactions ensures ongoing patient safety [40].

Additionally, being prepared to adjust orthodontic appliances or materials if hypersensitivity reactions occur helps ensure that treatment can continue with minimal disruption and discomfort for the patient [56].

However, in the event of an immediate hypersensitivity reaction, prompt medical intervention is necessary. For mild reactions, antihistamines and corticosteroids can be administered to alleviate symptoms. In cases of severe reactions, such as anaphylaxis, epinephrine should be administered immediately, and the patient should receive emergency medical care [56]. Additionally, removing the offending appliance or material is essential to prevent further exposure and exacerbation of the reaction.

### 9.2. Limitations, Strengths and Future Directions

Despite the intrinsic limitations of the article type, including selection bias, lack of comprehensiveness, absence of formal quality assessment, subjective interpretation, and challenges in addressing heterogeneity, the present narrative review may provide a comprehensive overview of the clinical features, diagnostic methods, and management strategies for oral manifestation of hypersensitivity reactions in orthodontic patients, also offering clinical insights and practical guidance that are directly applicable to clinical practice.

Future directions should focus on developing more biocompatible materials for fixed orthodontic appliances and identifying genetic markers that predispose individuals to hypersensitivity reactions. Advances in biomaterials science may lead to the development of novel alloys and coatings that minimize the release of allergenic ions, thereby reducing the incidence of hypersensitivity reactions in orthodontic patients [40]. Continued research and advancements in materials science and immunology will play a crucial role in enhancing the biocompatibility of orthodontic materials and reducing the incidence of hypersensitivity reactions, ultimately improving patient care and treatment outcomes [5].

## 10. Conclusions

Oral and extra-oral manifestations of (immediate and delayed) hypersensitivity reactions occur rarely and are due to the release of metal and non-metal ions from orthodontic appliances. They typically present as erythema, erosive–ulcerative lesions, and gingival hyperplasia, with histopathological findings showing inflammatory infiltrates.

Fixed orthodontic appliances pose a higher risk for oral manifestations of hypersensitivity reactions in orthodontic patients due to prolonged contact with oral tissues compared to removable appliances and clear aligners.

Nickel remains a significant allergen, and diagnostic tests like patch tests are essential for managing these reactions since early identification and removal of the allergenic material, combined with effective pharmacological and non-pharmacological treatments, can lead to complete resolution of symptoms and prevent recurrence. However, patients with a history of severe hypersensitivity reactions may require ongoing monitoring and may need to avoid certain materials in the future.

Proper patient education, regular monitoring, and the use of hypoallergenic materials are key strategies in ensuring the well-being of orthodontic patients. As a counterpart, a comprehensive understanding of the epidemiology, risk factors, and management strategies is essential for orthodontic practitioners to mitigate these reactions and ensure successful treatment outcomes. In this perspective, keeping dental and medical records updated during orthodontic treatment is crucial. Moreover, knowing a patient’s family history may help clinicians choose hypoallergenic materials and counsel patients about potential risks, and detailed inquiries about personal allergy histories, including the nature and severity of past reactions, may aid in predicting and preventing hypersensitivity issues.

Deepening the understanding of hypersensitivity epidemiology to materials like nickel, latex, acrylics, and other metals is essential for clinicians to select appropriate materials and mitigate risks. Ongoing research and advancements in materials science will enhance the safety and efficacy of orthodontic treatments, ultimately improving patient outcomes and satisfaction.

## Figures and Tables

**Table 1:** Allergy testing methods: field (medical specialties and hypersensitivity reaction investigated); mechanisms; process; advantages; and drawbacks.

Test Type	Field	Mechanism	Process	Advantages	Drawbacks
Patch Test [54]	Immunology Dermatology (Type IV—Delayed)	Relies on type IV hypersensitivity reactions. Sensitization occurs when antigen-presenting cells (APCs) capture and present allergens to T-helper cells, which multiply and travel back to the exposure site. Memory T-cells recognize the allergen upon re-exposure, triggering an immune response.	Application of allergens on the upper back using plastic or aluminum chambers. Patches remain for 48 h, followed by initial and subsequent readings at 72–96 h and sometimes at 7 days.	Identifies delayed-type allergic reactions not detected by blood or skin prick tests. Useful for chemicals causing contact allergic eczema.	Requires multiple appointments and careful monitoring. Some allergens require longer readings.
Skin Allergy Test [55]	Immunology Dermatology (Type I—Immediate)	Small amounts of allergens are introduced to the skin by pricking, scratching, injecting, or applying patches. Positive reactions are indicated by raised, red, itchy wheals.	Prick, scratch, or scrape tests involve introducing allergens to the skin, typically on the forearm. Positive reactions appear as raised, red, and itchy wheals.	Quick results for identifying allergies to pet dander, dust, pollen, foods, or dust mites. Minimal patient discomfort.	Some tests take several days for results. Risk of infection with scratch tests. False negatives possible.
Blood Test (RAST and ImmunoCAP) [56]	Immunology Dermatology (Type I—Immediate)	Detects specific IgE antibodies in the blood using radioimmunoassay. IgE binds to allergens, and radiolabeled anti-human IgE antibodies measure the level of allergen-specific IgE.	RAST involves binding allergens to an insoluble material, adding patient serum, and measuring IgE levels with radiolabeled antibodies. ImmunoCAP is a more advanced version with higher sensitivity and specificity.	High reproducibility and specificity. Suitable for patients on antihistamines or with widespread skin conditions. No need to stop antihistamine medication.	Longer processing time and higher cost. Less sensitive than skin tests. Potential for false positives due to cross-reactivity.
Basophil Activation Test [57]	Immunology Dermatology (Type I—Immediate)	Measures basophil response to allergens by detecting CD63 antigen expression on cell surfaces after activation. Basophils release histamine upon activation by IgE-bound allergens.	Basophils are stimulated with allergens, labeled with CD63 markers, and analyzed using flow cytometry. This method detects allergies like bee venom and drug allergies.	Uses minimal blood samples. Suitable for various allergies. Comfortable for patients.	Requires flow cytometry analysis. May need further validation for certain allergens.

## Data Availability

Data are freely available on PubMed/MEDLINE, Scopus, and Web of Science databases.
